# The Story of the Insane from Year to Year

**Published:** 1893-02-18

**Authors:** 


### THE STORY OF THE INSANE FROM YEAR TO YEAR.

(Fifth Series.)

### DERBY BOROUGH ASYLUM.

From the third annual report of this asylum we learn that 138 patients were admitted during the year; that 42 were discharged recovered, and that 47 died. The year closed with the names of 302 patients on the books. The percentage of recoveries was 43·7, and that of the deaths was 16·6. The recovery rate is high, and must be looked on as very creditable, considering the nature of the cases admitted during the year. Dr. Macphail points out that the admissions included 8 paralytics, 2 epileptics, 5 were congenital imbeciles, and a great many were in such feeble health that 11 per cent. of the total number died within the year.

Twenty-eight of the admissions were driven mad by drink, and 32 owed their affliction to hereditary tendency. Consumption was the cause of death in 3 cases, while brain diseases killed 27, and senile exhaustion 8. Dr. Macphail says—and he is right in saying it—that the importance of early treatment cannot be too frequently reiterated in asylum reports, and he backs up his remarks by showing that of his year's discharges the duration of the insanity "was under three months in 35 cases, under twelve months in 6 cases, and over twelve months in only 1 case." When speaking of the new Lunacy Act, Dr. Macphail says that further experience has not modified the unfavourable impression first formed of it. "It has imposed upon your staff a considerable increase of statutory duties, which are enforced with heavy (Dr. Macphail might have added needless) penalties, without any corresponding advantage to the patient, to the patient's relatives, or to the general public." Indeed, this Act has been of much worry and trouble to everyone, and of no advantage to anybody, unless, possibly, to the man who drafted the Bill. "The committee have had occasion to recognise the satisfactory manner in which Dr. Macphail has continued to discharge the responsible duties of his office."

### MIDDLESEX COUNTY ASYLUM AT WANDSWORTH.

A slight increase in the numbers took place here, there being on January 1st 1,089, and on December 31st 1,094. The admissions reached the respectable figure of 366, the recoveries 143, and the deaths 88. The recoveries are 41·7 per cent. on the admissions, and the deaths are 7 9 per cent. on the average number resident—the former being above and the latter below the average. Hereditary tendency and drink together are responsible for 100 of the year's admissions, and as 87 of the rest are put down as "unknown" it is certain that many more should be included among the hereditary cases. Twenty of the deaths were caused by consumption. From the Medical Superintendent's report we learn that an annexe for idiot children is about to be built, a step to which only one objection can be urged, namely, the already too large size of the asylum. When an institution contains 1,000 patients it cannot with advantage have another block tacked on to it unless, indeed, it be so small as to be useless for a populous county like Middlesex. We have asked before, and let us ask again, whether nothing can be done to induce the Secretary of State to refuse his sanction to these blunders. During the year the asylum lost by death its chaplain, its head female attendant, and one of its visitors. The head male attendant retired on a pension, and so did the laundryman. The visitors say "to Dr. Gardiner Hill their thanks are especially due for the efficient manner in which he has performed the heavy and responsible duties which devolve upon him." The weekly rate is 10s. 6d.

### CHESTER COUNTY ASYLUM AT UPTON.

The number of patients in this asylum at the beginning and end of the year was almost the same, being 604 on the 1st of January, 1891, and 602 on the last day of December. The admissions were 168, the recoveries 88, and the deaths 64. The recoveries work out at 52·3 on the admissions, and the deaths at 10·5 on the average number resident. Intemperance in drink is put down as the cause of insanity in 37 instances, and hereditary tendency in only 5, but we notice that "previous attacks" are given as the cause in 27. Consumption was fatal in only 4 cases. The asylum is full, and the Medical Superintendent says "that the time is very near at hand, if it has not already arrived, when some steps must of necessity be taken by those with whom the duty rests to make further provision for the proper care of the insane poor." It is curious to notice how almost universal it is for asylum committees to put off building until the place is so full that accommodation, often at a high rate, has to be found in other asylums. The only economical and humane course is to anticipate the wants of the county, and the visitors who have taken this step have always reaped the harvest while the others have had to pay, and often they have to pay as much to other counties as would have gone a long way towards making the additions to their own asylum. At Chester the visitors recognise the importance of the question, and they recommend that when further accommodation for the county will have to be provided, separate arrangements should be made for educating and training the pauper imbecile children. The visitors say it affords them "much pleasure to be able again to testify to the admirable and economical management of the asylum under their excellent superintendent and his staff." The weekly rate of maintenance is a fraction over 6s. 9d. per head, which we think is too low. The farm is credited with a balance of upwards of £400; but, as in so many asylum-farm accounts, we see nothing charged for rent, nor patients' labour, nor interest on capital, and the prices charged for goods supplied to the asylum are not given.

